# Application of multiple wrapped cancellous bone graft methods for treatment of segmental bone defects

**DOI:** 10.1186/s12891-019-2713-y

**Published:** 2019-07-27

**Authors:** Yunhong Ma, Sanjun Gu, Qudong Yin, Haifeng Li, Yongwei Wu, Zihong Zhou, Dehong Feng, Yongjun Rui

**Affiliations:** 1 Department of Orthopaedics, Wuxi the Ninth People’s Hospital Affiliated to Suzhou University, 999 Liangxi Rd, Wuxi, Jiangsu Province 214062 People’s Republic of China; 2 Department of Orthopaedics, Wuxi People’s Hospital, Wuxi, 214000 China

**Keywords:** Segmental long bone defect, Cancellous bone graft, Wrapped bone graft, Titanium mesh, Line mesh

## Abstract

**Background:**

The aims of this study were to discuss the principle, therapeutic effect and influencing factors of multiple wrapped cancellous bone graft methods for treatment of segmental bone defects.

**Methods:**

This study retrospectively analyzed the therapeutic effect of different wrapped autologous cancellous bone graft techniques on 51 patients aged (34.5 ± 11.5) years with segmental bone defects. Cancellous bones were wrapped with titanium mesh (*n* = 9), line mesh (*n* = 10), line suturing or line binding cortical block, (*n* = 13), or induced membrane (*n* = 19). The bone defeats were as follows: tibia (*n* = 23), radial bone (*n* = 10), humerus (*n* = 8), ulnar bone (*n* = 7), and femur (*n* = 3). The defect lengths were (5.9 ± 1.1) cm. The functionary recovery of adjacent joint was evaluated by the Paley‘s method and DASH, respectively.

**Results:**

The incision healed by first intention in 48 cases and secondary healing in 3 cases. All patients were followed up for 19.1 ± 7.1 (12–48) months. Other than one patient with nonunion who received a secondary bone graft, all the patients were first intention of bone healing (the healing rate was 98.0%). The healing time was 6.1 ± 2.1 (3–15) months. There were no significant differences in the healing time among the 4 groups (χ^2^ = 1.864, *P* = 0.601). The incidence of complications in the grafted site was 11.8%, whereas it was 21.6% in the harvest site. At the last follow-up, all the patients had recovered and were able to engage in weight-bearing activities. The functional recovery was good to excellent in 78.4% of cases, there were no significant difference among the 4 groups (χ^2^ = 5.429, *P* = 0.143).

**Conclusions:**

Wrapped cancellous bone grafting is a modified free bone graft method that can be used in the treatment of small and large segmental bone defects as it prevents loosening and bone absorption after bone grafting. The effect of bone healing is related with the quality and quantity of grafted bone, stability of bone defects, property of wrapping material and peripheral blood supply.

## Background

Bone defects, which are commonly induced by bone infection, removal of bone tumors, and severe trauma, account for 15% of fractures of limbs and cause a decline in the quality of life of patients. Bone defects longer than 2 cm will not heal by themselves and need to be repaired. A free bone graft is the “gold standard” for the repair of bone defects [1–4]. Traditionally, a free cancellous bone graft was considered suitable only for segmental bone defects less than 4–6 cm and unsuitable for defects more than 4–6 cm due to frequent bone absorption, which resulted in a high rate of nonunion [1–4]. Although multiple methods, such as the Ilizarov technique, vascularized bone graft, blood donor bone grafting, and large allograft bone grafts, are emerging that appear to be suitable for segmental long bone defects greater than 4–6 cm, all these methods have various deficiencies [5, 6]. For example, the Ilizarov technique has shortcomings of reduced self-healing rates, long fixation times, and a high incidence of complications, such as joint stiffness, infection, and loosening of the nail path. Vascularized bone grafting requires microsurgical techniques, which result in difficult to be popularized. Besides, the surgical trauma is too large. The shortcomings of large allogeneic bone grafts include slow healing time, high rates of refractures and infections, and foreign body reactions, all of which restrict its widespread application. Tissue engineering techniques are an option but are only in the experimental research stage [5, 6].

In the last two decades, many improved free bone graft methods have been proposed to treat segmental long bone defects. For example, in 2000, Masquelet [5] reported a wrapped cancellous bone graft using the induced membrane in the treatment of 35 cases with long segmental bone defects of 4–25 cm. All the patients obtained clinical healing after an average of 4 months. In the same year, Cobb et al. [6] reported a wrapped bone graft using titanium mesh in the treatment of large segmental bone defects. Later, Tian Wen et al. [7] adopted a line mesh and line suture or line-binding cancellous bone graft to treat segmental long bone defects and obtained satisfactory effects. All the aforementioned methods employ cancellous bone wrapped in a mechanical device, thereby overcoming the shortcomings of traditional cancellous bone grafts [7]. These improved free bone graft methods change the traditional view of the unsuitability of free bone graft as a treatment for large segmental one defects [5, 6, 8–10]. Thus, modified free bone graft is becoming an effective method for the treatment of segmental bone defects. We named this method the “wrapped bone grafting technique”. Recently, the development of a new wrapped device-absorbed mesh with holes and progress in the harvesting of large volumes of nonstructural autogenous bone have advanced the development of wrapped bone grafting techniques [8, 9].

Few literatures reported the influence factors of wrapped bone grafting and the comparison of therapeutic effects of different wrapped bone grafting methods. From 2007, we started to adopt various methods of wrapped cancellous bone grafting to treat segmental bone defects. In this study, we investigate the principle, therapeutic effect and influencing factors of wrapped cancellous bone graft for bone defects.

## Methods

### Inclusion and exclusion criteria for cancellous bone wrapped grafting cases

The inclusive criteria were as follows: (1) a segmental long bone defect in one of four limbs; (2) a bone defect longer than 3 cm; (3) aged 13–73 years; (4) Infected bone defects were got controlled for at least 3 months; and (5) suitable clinical and imaging data.

The exclusion criteria were as follows: (1) follow-up time of less than 12 months; (2) poor patient compliance; (3) pathological bone defect; (4) poor skin condition, with no visible improvement; and (5) poor blood supply, with or without nerve damage and unsuitable for limb salvage.

### General data

This study consisted of 51 patients (32 males and 19 females) aged 34.5 ± 11.5 years old (ranged 13–69). In terms of the causes and types of segmental bone defects, in 28 cases, they were the result of trauma (noninfected bone defect). In 23 cases, they were due to infection after a fracture merge or osteomyelitis following debridement (infected bone defect). All infected bone defect cases had symptoms of swelling, fever, and pain. Among these cases, pus was present in the wounds in 19 cases, and a sinus canal was present in 6 cases. All cases of infective bone defects were examined with bacterial culture of secretions, of which 16 were positive. The locations of the bone defects were as follows: tibia (*n* = 23), radial bone (*n* = 10), humerus (*n* = 8), ulnar bone (*n* = 7), and femur (*n* = 3). The mean defect length was 5.9 ± 1.1 cm (ranged 3–9). The mean time between the diagnosis of the bone defect and bone graft treatment was 3.34 ± 1.82 months (8 d to 9 months). In terms of the wrapped devices, titanium mesh (*n* = 9), line mesh (*n* = 10), line suture or binding cortical block (*n* = 13), and induced membrane (*n* = 19) materials were used. Nine patients were combined with other fractures or injuries. General information on the patients is shown in Table 1.

**Table 1:** General data for four cancellous bone wrapping grafting techniques

Groups	Cases	Age (Year)	Sex (Male/Female)	Bone defeat and infection (cases)		Length (cm)	Interval between defect and graft (Months)
Titanium mesh	9	35.1 ± 11.7	7/2	Trauma		6.5 ± 0.5	2.2 ± 0.9
Line mesh	10	33.4 ± 10.8	6/4	Trauma/infection	6	6.0 ± 1.1	3.2 ± 0.8
Line-binding cancellous bone	13	34.5 ± 10.6	7/6	Trauma		6.5 ± 0.7	1.9 ± 0.8
Induced-membrane	19	36.0 ± 13.1	11/8	Trauma/infection	17	5.1 ± 1.0	4.9 ± 1.8

This study was approved by the ethics committee of Wuxi the Ninth People’s Hospital Affiliated to Suzhou University and Wuxi People’s Hospital, and all patients provided signed informed consent.

### Surgical methods

Before bone grafting, all the patients with noninfectious bone defects combined with skin defects underwent skin flap or free skin grafting to repair the wound. The time of wound healing was at least 4 weeks.

**Fig. 1:**
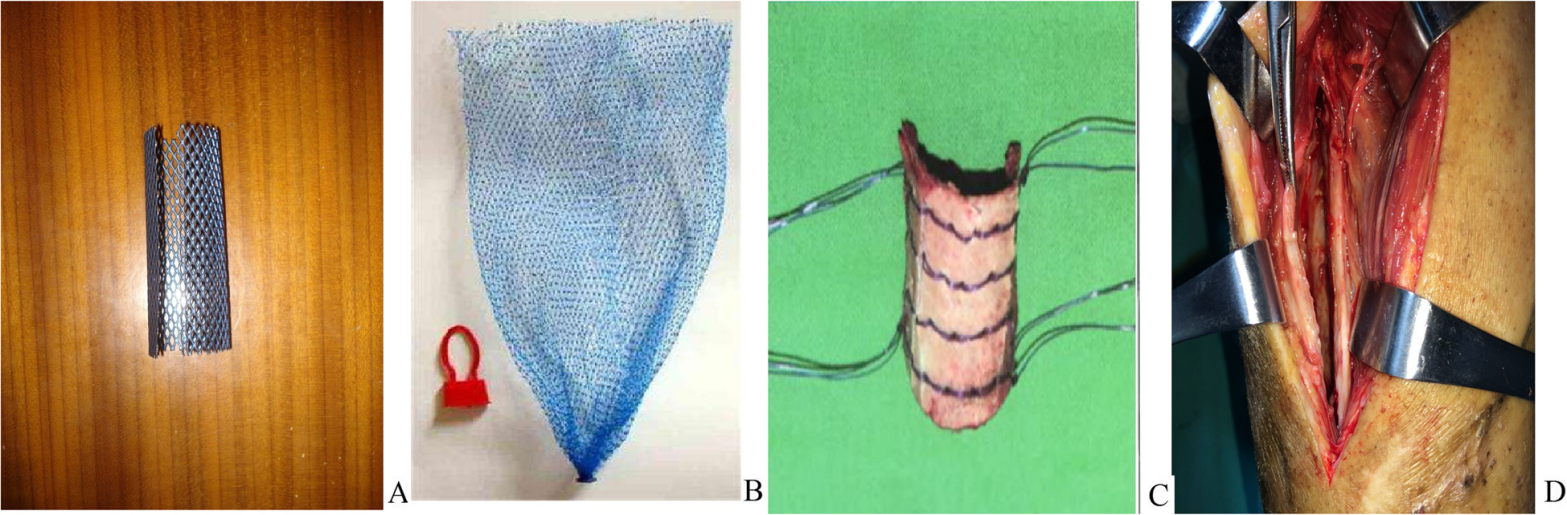
Graphical representation of the wrapped materials. **a** Titanium mesh, (**b**) line mesh (**c**) line suturing or line binding, and (**d**) induced membrane

The operative methods were as follows: First, a longitudinal incision was made to perform debridement, ensuring a blood supply for the broken bone. Second, bone fixation was done using established methods, such as an intramedullary nail, a locking plate, and external fixator. Third, the bone defects were treated using the wrapped cancellous bone grafting technique with one of four different materials. In the titanium mesh cases, titanium mesh of appropriate length and width was cut and rolled into a cylindrical object. This was used to wrap the cancellous bone and a small amount of cortical bone, fixing on two ends of the defect [11]. In the line mesh cases, a surgery operator wrapped the two ends of the defect with the self-designed line mesh and then put the cancellous bone in the center of the mesh, with a small amount of cortical bone or cortical bone in the outer layer before tightening the mesh [10]. In the line suturing cases, the free, bloodless cortical bone mass and thin layer of cortical bone of the iliac bone were connected by drilling and suturing with lines and then placed in the defect. In the line binding cases, the above cortical bone was cut into thin strips and then placed in the bone defect beneath multiple lines. The cancellous bone or with a small amount of granular cortical bone was put in the inner of different wrapping device and impacted to a degree. Finally, tighten the wrapping device. In the induced membrane cases, cancellous bone and a small amount of cortical bone were implanted into the induced membrane, and then suture the opened induced membrane. All the materials are shown in Fig. 1.

In the bone mass and bone harvesting operation, the size of the bone graft material was 1.5–2.0 times the mass of the bone defect for titanium mesh. The size of bone graft material was 1.5 times that of the bone defect for the line mesh, line suturing, line binding, and induced membrane. In cases where the amount of autologous cancellous bone was insufficient, it was mixed with no more than one-quarter of cortical bone and/or artificial bone. The first choice for autologous cancellous bone extraction was the posterior region of the iliac crest, then proximal tibia was considered as there were abundant autologous cancellous bone. The anterior region of the iliac crest was used to extract autologous cancellous bone and incidental cortical bone in cases where the posterior region was not sufficient.

### Postoperative treatment

For infection prevention, antibiotics were given for 3–5 days postoperatively. None of the patients required external fixation with a cast or brace. Rehabilitation activities started 3 days postsurgery. The gradual partial weight bearing was permitted after 2 weeks, and complete weight bearing was allowed when bone connection was noted by a radiological examination. All the patients underwent an X-ray examination each month until bone healing. Patients with unclear X-ray examination (such as in titanium mesh group) were evaluated by CT imaging. After bone healing, the follow-up interval was 2–3 months. Six months after bone healing, the follow-up interval was 4–6 months. Internal fixation may be removed 1 year after clinical healing.

### Evaluation of efficacy

Clinical healing was determined by imaging of bone healing and clinical manifestations. In the imaging studies, bone healing was judged as the formation of continuous bone callus between the ends of the defect. The functionary recovery of lower limbs was evaluated by the Paley‘s method [12], whileas the functionary recovery of upper limbs was evaluated by DASH.

### Statistical analysis

Statistical analysis was performed using SPSS 16.0 statistical software. Data were presented as mean ± SD. The comparison of measurement data between the two groups was performed with two independent samples *t* tests. Counting data were compared using a chi-square analysis, and the four-grid table method was used for comparisons of rates. A value of *P* < 0.05 was considered statistically significant.

## Results

### General information on the fixation methods and bone harvesting sites

In terms of the fixed methods used, there were 25 cases of intramedullary nail fixation, 22 cases of locking plate fixation, and 4 cases of external fixator. With regard to bone harvesting sites, bone was obtained from the unilateral iliac posterior crest and ipsilateral iliac anterior crest (*n* = 13), unilateral iliac posterior crest and ipsilateral proximal tibia bone (*n* = 11), bilateral iliac posterior crest (*n* = 15), bilateral iliac posterior crest and unilateral iliac anterior crest (*n* = 7), and bilateral iliac posterior crest, unilateral iliac anterior crest, and unilateral tibia proximal (*n* = 5).

### Surgical healing and functionary recovery of adjacent joint

**Fig. 2:**
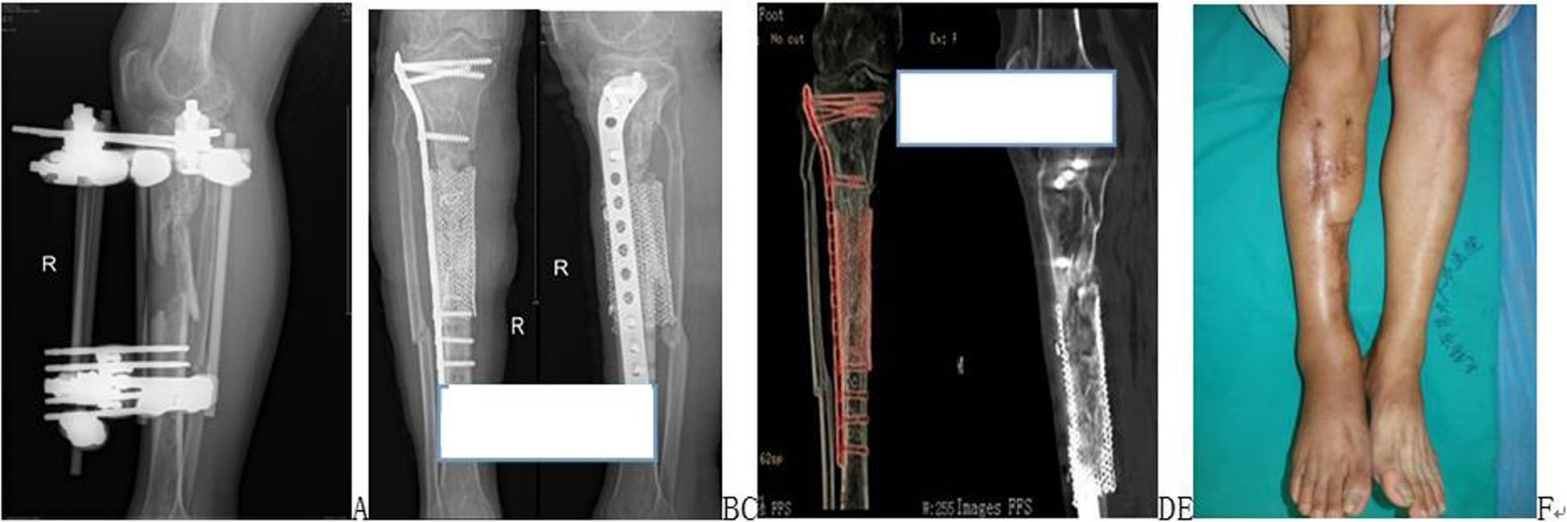
A patient with right tibial fractures combined with bone defects treated with titanium mesh wrapped cancellous bone grafting. **a** Preoperative X-ray films. **b**–**e** Postoperative X-ray films and CT show healing. **f** Postoperative appearance 1 year postsurgery

**Fig. 3:**
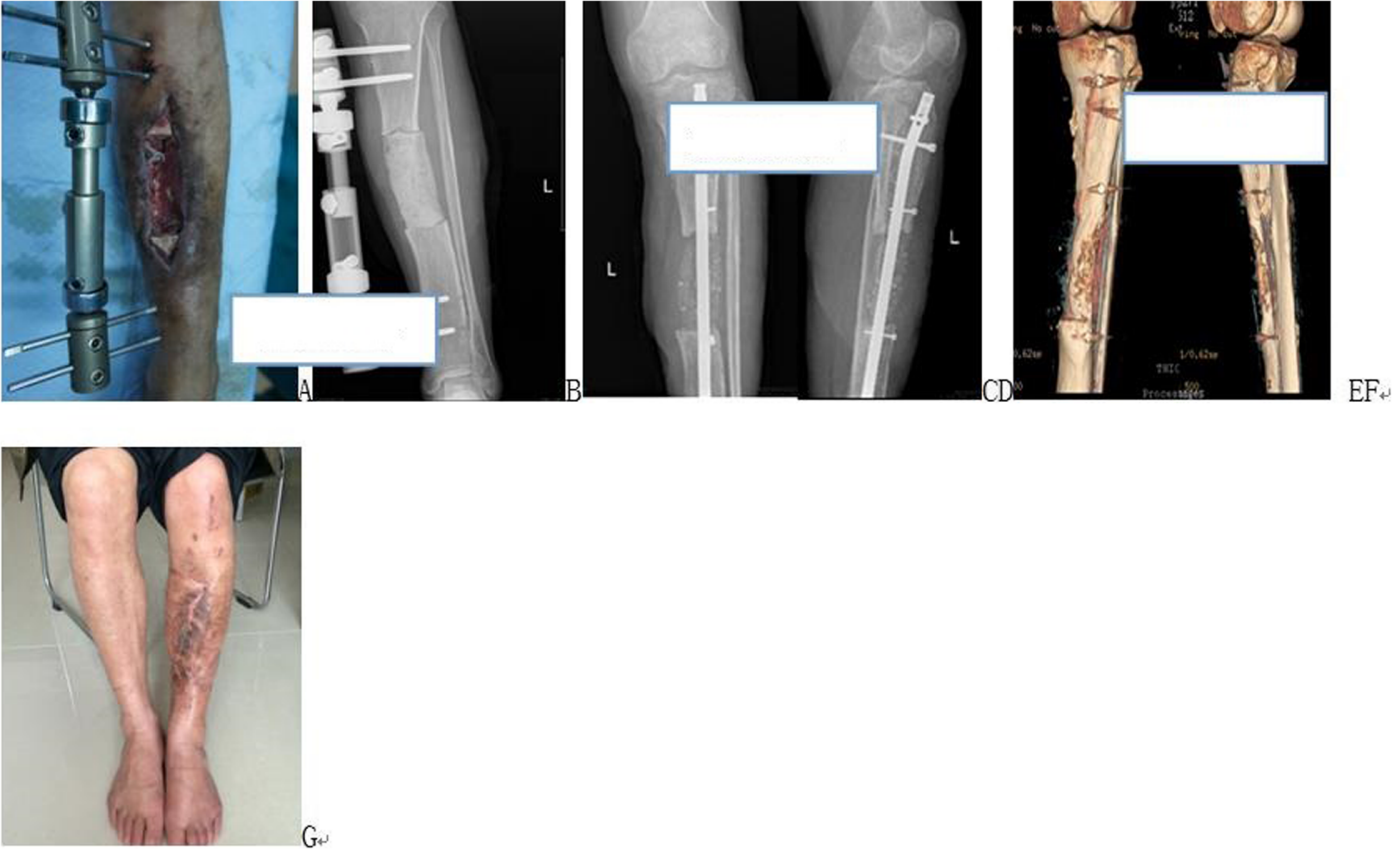
A patient with an infective left tibial bone defects who was treated with induced membrane cancellous bone graft. **a** Preoperative appearance. **b** Postoperative X-ray films after skin flap repair and bone cement packing. **c** and **d** Postoperative X-ray films 3 months postsurgery. **e** and **f** CT three-dimensional reconstruction to evaluate bone healing 1 y postsurgery. **g** Appearance 1 year postsurgery

**Fig. 4:**
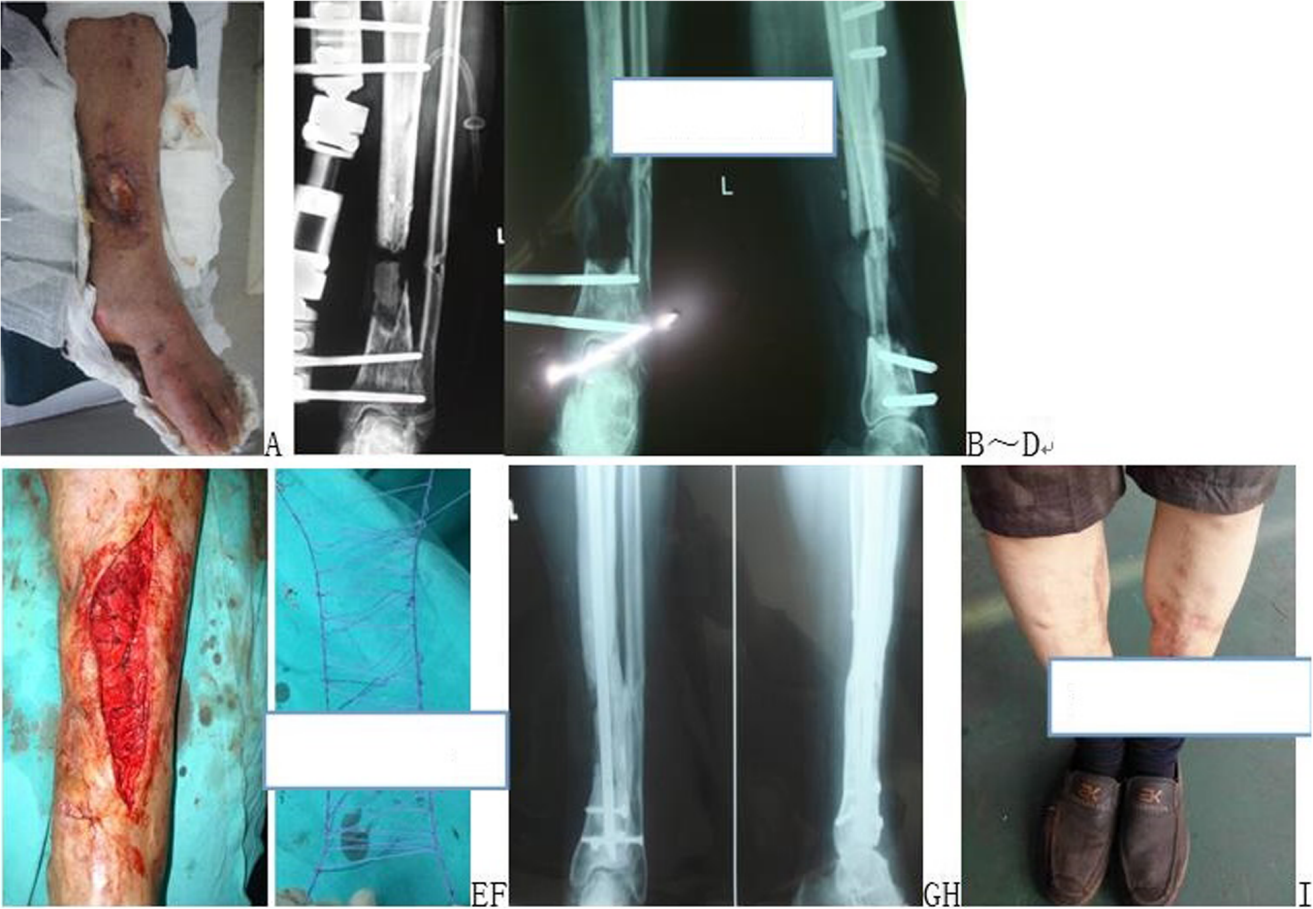
A patient with infected left tibial bone defects treated with line mesh wrapped cancellous bone graft in second intention surgery. a Appearance of the bone defects. **b**-**d** Preoperative X-ray films. **e**, **f** Surgical procedure using the line mesh material and the wrapped cancellous bone graft technique. **g**, **h** Appearance 1 year postsurgery. **i** Appearance of bone healing one year after bone grafting

**Fig. 5:**
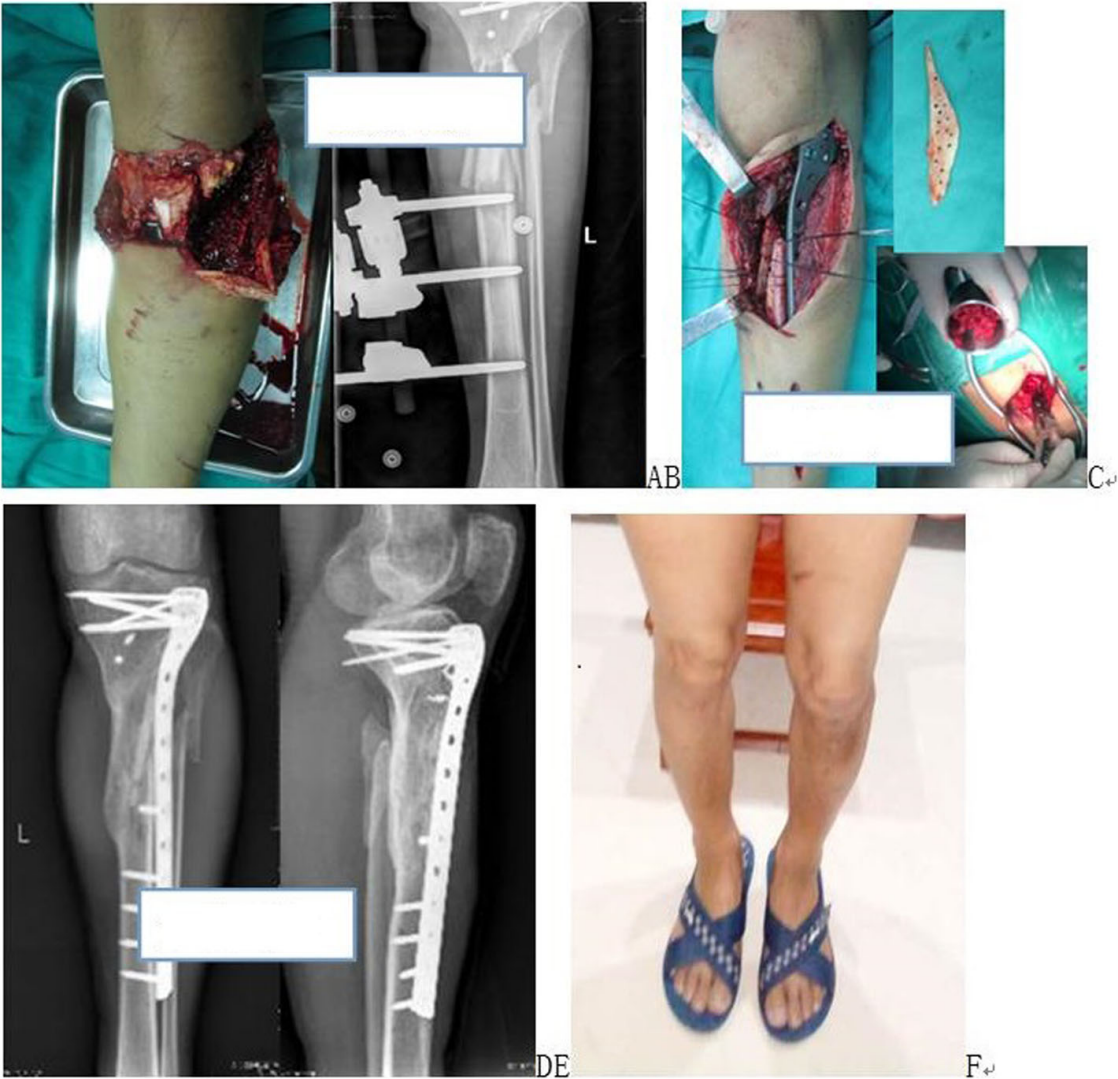
A patient with open upper tibial fractures combined with bone defects treated with line binding cortical bone wrapped cancellous bone graft. **a** and **b** Wound appearance and X-ray films of external fixation and reserved free cortical bone. **c** Surgical procedure using line binding cortical bone and the wrapped cancellous bone graft technique in second intention surgery. **d** and **e** X-ray films show bone healing preoperative 10 months. **f** Appearance 18 months postsurgery

In terms of incision healing, 48 cases were first intention healing, and 3 cases were second intention healing. There were no significant differences in the incision healing among the four groups (χ^2^ = 1.931, *P* = 0.587). All the patients were followed up for 19.1 ± 7.1 months. With regard to bone healing, in one case in the induced membrane wrapped group, a secondary bone graft (healing time: 15 months) was required. The others were first intention of bone healing (the healing rate was 98.0%). The overall clinical healing time was 6.1 ± 2.1 months. There were no significant differences in surgical incision healing times among the four groups. Figure 2 shows a bone defect treated with the wrapped bone grafting technique using titanium mesh, in which the average healing time was 5.44 months. Figure 3 depicts a bone defect treated with the wrapped bone grafting technique using an induced membrane, where the average healing time was 6.10 months. Figure 4 shows a bone defect treated with the wrapped bone grafting technique using a line mesh, and the average healing time was 6.50 months. Figure 5 presents a bone defect treated with the wrapped bone grafting technique using line suturing or line binding, in which the average healing time was 6.23 months. There were no significant differences in the healing among the four groups (*χ*^*2*^ *= 1.864, P = 0.601*). By the end of the follow-up, all the patients had recovered and were able to engage in weight-bearing activities.

In terms of the functionary recovery, it was classified as excellent in 18 cases and as good, fair, and poor in 22, 8, and 3 cases, respectively. Thus, the rate of good to excellent functional restoration was 78.43%, there were no significant difference among the 4 groups (χ^2^ = 5.429, *P* = 0.143). The reasons for poor or fair functional recovery of the affected limb were due to the limitation of functional activities of the adjacent joints. There was no fixator broken or refractures after bone healing. In patients with infective bone defects, there were three cases of postoperative recurrence of infection (one case in the line mesh group and two cases in the induced membrane group). The infection in one case of the induced membrane group was controlled with conservative treatment. The other two cases required surgical intervention to control the infection. No postoperative infections or recurrence of infections occurred in any of the other patients (Table 2).

**Table 2:** Therapeutic outcome of four cancellous bone wrapping grafting techniques

Groups	Cases	Healing	Healing time (M)	Complication of graft (cases)	Complication of taking bone (cases)	Recovery (excellent/good/fair/poor)
Titanium mesh	9	9/9	5.4 ± 1.1	1	2	8/1/0/0
Line mesh	10	10/10	6.5 ± 2.0	1	2	6/2/2/0
Line-binding cancellous bone	13	13/13	6.2 ± 1.7		3	6/4/2/1
Induced-membrane	19	18/19	6.1 ± 2.6	4	4	9/4/4/2

### Complications

The incidence of complications in a grafted site bone grafting was 11.8% (two cases of healing by second intention, one case of nonunion, and three cases of infection recurrence). The nonunion was healed after regrafting. Among the patients with recurrent infection, 1 case needed surgery and the other 2 cases needed conservative treatment to control the infection. The incidence of complications, such as pain and numbness in harvesting site, was 21.6% (11/51). The pain visual analogue scale score was 1.5 points (range: 1–3) (Table 2).

## Discussion

The present study demonstrated that, as a modified free bone graft method, the wrapped cancellous bone grafting avoids or obviously decreases loosening of grafted bone and bone absorption, promotes the rapid healing and high healing rates and is suitable for both small and large segmental bone defects.

Cancellous bone has characteristics of bone induction, and osteogenesis and is regarded as the best bone graft material. The main reason of traditional free cancellous bone graft was considered unsuitable for large segmental bone defects is due to a high rate of failure caused by loosening and absorption resorption of the grafted bone as lack of wrapped device, the grafted bone is vulnerable to vibration from the surrounding tendons or muscles and body vibration, resulting in loosening or sliding, even bone absorption [6]. For this reason, some authors suggest the use of a plaster cast or brace for a period of time postoperatively. However, immobilization cannot prevent bone absorption. Furthermore, it hampers functional recovery and bone healing.

As compared with the traditional method, the wrapped cancellous bone grafting improves both the volume and harvesting method of grafted cancellous bone [6], which overcomes the shortcomings of traditional free bone graft method. First, the technique utilizes mechanical packing device, which fixes the bone graft material and avoids undesirable stimulation of surrounding tendons or muscles and vibrations of the bone graft material [3, 13]. Second, the wrapped bone graft technique requires abundant autologous cancellous bone [6]. In the technique using titanium mesh, the amount of grafted bone is 1.5–2.0 times that of the bone defect that has to be filled. The amount of grafted bone is 1.5 times that of the bone defect for line mesh, line suturing or binding, and induced membranes. If the amount of autogenous cancellous bone is insufficient (e.g., in cases of large bone defects), cancellous bone can be mixed with cortical bone or artificial bone, but no more than one-quarter of cortical/artificial bone can be used [14, 15].

Harvesting methods of autologous cancellous bone have also made great progress. Studies have also shown that more cancellous bone can be obtained from the posterior iliac crest than from the anterior iliac crest, therefore the former is the first choice [7, 16]. The proximal tibia is also considered a rich source of cancellous bone, with few complications [5, 6, 14, 16]. Based on our experiences, autologous cancellous bone can be harvested from multiple sites to meet the need for large amounts of cancellous bone graft. For example, the amount of autogenous cancellous bone from the bilateral iliac crest mixed with attached cortical bone can fill a 1.5 measuring cup, which can meet the needs of a 6.0-cm-long tibial shaft defect. Autogenous cancellous bone from the bilateral proximal tibia can fill one cup. Using autogenous cancellous bone from the aforementioned four sites, sufficient graft material to meet the needs of a 9.0–10.0 cm-long tibial shaft defect can be obtained. By using a reamer-irrigator-aspirator (RIA), a large amount of granular autologous bone graft material can be collected from the femur or tibial medullary cavity [8, 9]. The amount of granular autologous bone grafts collected from a unilateral femur using an RIA can be up to 40–90 cm^3^ (average of 67 cm^3^), which is more than the average volume of 26 cm^3^ in the anterior iliac crest and 36 cm^3^ in the posterior iliac crest. Bone graft material from a bilateral femur obtained by an RIA can meet the needs of a tibial bone defect longer than 10.0 cm [8]. Furthermore, the bone graft material collected by an RIA contains a large amount of cancellous bone and cortical bone granules. These are rich in osteoblasts, marrow stromal stem cells, fibroblast growth factors, platelet-derived growth factors, insulin-like growth factors, bone morphogenetic protein-2, and transforming growth factor beta 1 and have the same osteogenic effect as ilium [8, 9]. As a result, RIA provides an effective method of bone harvesting for large segmental bone defects treated using the wrapped bone graft technique.

An additional advantage of the wrapped bone graft technique is that patients can take early postoperative rehabilitation exercise due to the effect of wrapping on the bone graft material and reliable internal or external fixation [8, 17–21]. Early rehabilitation is helpful to stimulate bone healing and functional recovery of the joint. In addition, the wrapped device plays an important role in vascularization and osteogenesis of the grafted bone because it has holes and good biological properties. The holes provide a pathway for new blood vessels and osteogenic factors. Therefore, the grafted bone is nourished by blood vessels and osteogenic factors from the surrounding area and forms new bone. In terms of the different wrapping materials, titanium mesh has good biocompatibility and some degree of a bone inductive effect [6]. The induced membrane has good osteogenic properties and a blood supply, especially in the early stage when it has a rich vascular system and can secrete osteogenic growth factors (e.g., transforming growth factor beta 1 and bone morphogenetic protein-2) and angiogenic growth factors (e.g., CD31+ endothelial cells, vascular endothelial growth factors, and osteogenic precursor cells) [16, 17, 19, 22]. The availability of RIAs overcomes previous difficulties in harvesting of rich autologous bone and makes it possible to obtain a large amount of autologous cancellous bone. Recently, an absorbable mesh for wrapped cancellous bone grafts to treat bone defects has become available [23]. The use of this absorbable mesh can shorten the operation time and further promote the popularity of wrapped bone graft technique.

Cobos et al. [6] reported two cases of 8.5–9.5 cm defects of tibia bone. Attias et al. [13] reported three cases of tibial bone defects, where the average defect length was 12.2 cm. Ostermann [20] reported one case of a segmental tibial bone defect. Attias et al. [21] reported one case of an 8-cm humerus bone defect. In these cases [6, 13, 21, 22], the defects were treated with titanium mesh wrapped cancellous bone graft, and bone healing was achieved 1 year postsurgery. Karger et al. [14] reported 84 patients with long bone defects, with the longest being 23 cm, that were treated with an induced membrane wrapped autologous cancellous bone graft. They reported a healing rate was 90%. McCall et al. [9] reported 21 cases of bone defects of the lower limb (an average of 6.6 cm) treated with an induced membrane wrapped bone graft by RIA. In their study, 20 cases obtained bone healing, and one case was lost to follow-up. Apard et al. [17] reported 12 patients with tibial bone defects (average length of 8.7 cm) that were treated with an induced membrane wrapped bone graft and achieved a bone healing rate of 91.6%. Whately et al. [8] reported one case of a 10-cm-long tibial defect treated using an absorbable polymer mesh wrapped bone graft by RIA plus intramedullary nail fixation. They reported clinical healing of bone 6 months postoperatively. Liu Yao-xi [7] reported congenital tibial pseudarthrosis in 12 pediatric patients with bone defects (4–11 cm) treated with a line suturing cortical bone wrapped cancellous bone graft and achieved 100% healing. In the present study, the average bone defect length was 5.9 cm, the longest tibia defect was 9 cm, and the longest humerus defect was 7 cm. The average healing time in the patients treated with the four different materials using the cancellous bone graft method was 6.1 months, and the healing rate was 98%. The incidence of complications in the grafted area was 11.8, and 21.6% in the harvesting site, which are not higher than other methods [1, 5, 6, 9]. Our data indicate that all four materials are effective for the treatment of segmental bone defects. They were associated with accelerated healing, high rates of healing, and few complications, irrespective of the length of the bone defect. Notably, the titanium mesh had the shortest healing time (average of 5.44 months), pointing to its superiority over the other wrapped materials. In theory, the healing effect of the induced membrane wrapped bone graft should have been the best, as the induced membrane has a mechanical package and fixation effect and osteogenic induction. As reported in the literature [3, 5, 22], the shortest healing time using the induced membrane technique for the treatment of bone defects was 3 to 4 months, and the longest healing time was 6–10 months after grafting. In the present study, 89.5% of the patients who were treated with the induced membrane wrapped bone graft were infected defects. In all the patients, the infection was controlled at least more than 3 months before bone grafting. The time from bone cement filling to bone grafting averaged 5.1 months in our study. Previous studies showed that the best osteogenic activity of an induced membrane after bone cement filling occurred 4–6 wk. after filling and that the osteogenic activity declined gradually from then on [16, 17, 19, 22]. In the present study, in the induced membrane group, the osteogenic activity was reduced and the blood supply was poor in 5 months after bone cement filling, and the induced membrane exhibited only mechanical and fixation effects. In addition, in this study, one patient had nonunion and required a secondary bone graft, in which the clinical healing time was 15 months. Thus, the induced membrane group was characterized by a relatively long healing time and high rate of complications.

As stability is the main factor affecting bone healing, reliable internal fixation must be chosen. According to our experience, intramedullary nails should be the preferred method of internal fixation for long bone shaft defects. This is because they have good biomechanical stability and save bone graft materials by occupying the position of the medullary cavity. If one side of the medullary cavity is large, the blocking nail technique should be used to prevent the end instability (Fig. 3c). Plate fixation is preferred for epiphyseal fixation. If the bone defect is close to the joint surface, internal fixation is difficult. In such cases, external fixation is a good choice [16].

Li Lin et al. [24] reported four cases of large segment bone defects of the tibia treated with cortical bone graft wrapped by an induced membrane. The nonunion rate was 50%, and bone healing of nonunion was achieved after the cortical bone graft was replaced with cancellous bone graft. In the present study, one case of bone nonunion occurred because the volume of bone cement filling at the ends of bone defect was too small and the induced membrane volume that formed was also small, which resulted in less bone graft and insufficient bone connection. The reasons for slower healing of the middle and lower segments of the tibia are a weak blood supply and coarser bone. Therefore, the quality of bone grafting, fracture stability, wrapped material properties, and peripheral blood supply are the main factors influencing the efficacy of bone healing by cancellous bone grafting.

The selection of different wrapped bone graft methods depends on the specific situation of the bone defects. For bone defects in location of non weight-load such as upper limbs, line mesh is the preferred method. If there are multiple free cortical bone blocks, the preferred method is line suturing or binding. For large segmental defects of long lower limbs, titanium mesh is preferred because of its reliable fixation and rich bone graft material can be filled [10]. For open and infected bone defects, induced membrane technique is indicated [16]. Irrespective of which wrapped method is chosen, fixation using an intramedullary nail is the first choice. For large segmental bone defects, RIA is the preferred way to harvest bone. If RIA is not available, the preferred site to harvest bone is the posterior iliac. If the quantity harvested from the posterior iliac cannot meet the need, then the proximal tibia can be selected. If the quantity of harvested aotogenous bone is insufficient and cannot meet the need, then allogeneic or artificial bone can be addited.

There are some deficiencies in performing wrapped cancellous bone grafting. First, it requires good conditions of soft tissue [5, 16]. In bone defects accompanied by soft tissue defects, the wound repair with skin flap must have no tension, the skin scar near the bone defect should be removed and replaced with a skin flap. In our study, the incision in one case was disrupted because of high tension of the skin flap and required another operation Therefore, in cases of poor skin condition, the wrapped cancellous bone graft technique is not suitable. A more suitable choice in such cases is Ilizarov technique. Second, the infection must be controlled and keep a normal state in erythrocyte sedimentation rate and C-reactive protein level for more than 3 months before the bone graft [6, 7]. Third, complications of harvesting large amount of graft materials from the iliac crest remain an unsolved problem. A previous study reported that the incidence of complications (pain and numbness) in the anterior iliac crest varied from 6 to 36% [9]. In the present study, the incidence was 21.6%. Research also reported that average intraoperative blood loss when using RIA to harvest bone graft materials was 674 ml and that iatrogenic fractures may occur when harvesting a large amount of bone graft material [25]. In addition, another study reported that although callus formation was fast, corticalization of the callus was very slow and usually took 2–3 y [14]. Thus, stress fractures can occur before corticalization [14]. Finally, the removal of titanium mesh and plates after a bone graft is difficult [13].

In this paper, only four kinds of wrapped bone grafting methods were used in the earlier stage. This study did not include fascia, including femoral fascia, and absorbable mesh that have begun to be used recently in clinical applications. This paper was a retrospective clinical study, and only a few patients were treated with each of the wrapped materials. Thus, there is sampling error. Moreover, there were large baseline differences in the preoperative general data. Furthermore, there was bias, which was not suitable for the statistical analysis to compare the differences in the various wrapped methods. Therefore, more clinical data from multicenter studies, large samples, and experimental research are needed compare differences in bone healing and functional recovery using the various wrapped graft methods and confirm the therapeutic effect of these methods.

## Conclusion

Our current results suggested that wrapped cancellous bone grafting is a modified free bone graft method that can be used in the treatment of small and large segmental bone defects as it prevents loosening and bone absorption after bone grafting.

## Data Availability

The authors declare that all data supporting the findings of this study are available within the paper and its supplementary information files, or are available from the corresponding author upon reasonable request.

## Abbreviations

BMP-2: Bone morphogenetic proteins
RIA: Reamer-irrigator-aspirator
TGF-beta 1: Transforming growth factor beta 1
